# Annual cost of antiretroviral therapy among three service delivery models in Uganda

**DOI:** 10.7448/IAS.19.5.20840

**Published:** 2016-07-20

**Authors:** Lung Vu, Samuel Waliggo, Brady Zieman, Nrupa Jani, Lydia Buzaalirwa, Stephen Okoboi, Jerry Okal, Nagesh N Borse, Samuel Kalibala

**Affiliations:** 1 Population Council, Washington, DC, USA; 2 Kitovu Mobile AIDS Organisation, Masaka, Uganda; 3 Uganda Cares, Kampala, Uganda; 4 The AIDS Support Organisation, Kampala, Uganda; 5 Population Council, Nairobi, Kenya; 6 US Agency for International Development, Washington, DC, USA

**Keywords:** ART, cost, efficiency, task-shifting, community-based ART, Uganda

## Abstract

**Introduction:**

In response to the increasing burden of HIV, the Ugandan government has employed different service delivery models since 2004 that aim to reduce costs and remove barriers to accessing HIV care. These models include community-based approaches to delivering antiretroviral therapy (ART) and delegating tasks to lower-level health workers. This study aimed to provide data on annual ART cost per client among three different service delivery models in Uganda.

**Methods:**

Costing data for the entire year 2012 were retrospectively collected as part of a larger task-shifting study conducted in three organizations in Uganda: Kitovu Mobile (KM), the AIDS Support Organisation (TASO) and Uganda Cares (UC). A standard cost data capture tool was developed and used to retrospectively collect cost information regarding antiretroviral (ARV) drugs and non-ARV drugs, ART-related lab tests, personnel and administrative costs. A random sample of four TASO centres (out of 11), four UC clinics (out of 29) and all KM outreach units were selected for the study.

**Results:**

Cost varied across sites within each organization as well as across the three organizations. In addition, the number of annual ART visits was more frequent in rural areas and through KM (the community distribution model), which played a major part in the overall annual ART cost. The annual cost per client (in USD) was $404 for KM, $332 for TASO and $257 for UC. These estimates were lower than previous analyses in Uganda or the region compared to data from 2001 to 2009, but comparable with recent estimates using data from 2010 to 2013. ARVs accounted for the majority of the total cost, followed by personnel and operational costs.

**Conclusions:**

The study provides updated data on annual cost per ART visit for three service delivery models in Uganda. These data will be vital for in-country budgetary efforts to ensure that universal access to ART, as called for in the 2015 World Health Organization (WHO) guidelines, is achievable. The lower annual ART cost found in this study indicates that we may be able to treat all people with HIV as laid out in the 2015 WHO guidelines. The variation of costs across sites and the three models indicates the potential for efficiency gains.

## Introduction

Although notable progress has been made in the provision of HIV and AIDS services, the need for HIV and AIDS services continues to expand faster than the available resources in low- and middle-income countries. In the case of Uganda, coverage of the population in need of antiretroviral therapy (ART) was estimated at 331,000 in 2014, representing only half of the total number of people living with HIV (PLHIV) [1]. ART offers PLHIV a chance to live a normal lifespan. Consequently, HIV is increasingly seen as a chronic illness rather than an acute epidemic [2–4]. In addition, new HIV infections continue to occur, contributing to an increased cumulative number of PLHIV [4]. Furthermore, ART has been increasingly seen as an important prevention strategy (treatment as prevention). The World Health Organization (WHO) has recently recommended HIV treatment to all PLHIV regardless of their CD4 count, and many countries are planning on adopting this recommendation [3]. All of these factors are contributing to an increased demand on human and financial resources to deliver ART, thus careful planning and budgeting are needed to ensure universal access to ART [4,5].

The increased demand on human and financial resources to scale up ART presents a problem as many low income countries have historically experienced severe health worker shortages [5]. For example, in Uganda the ratio of doctors to patients is 1:22,000, suggesting an 80% overall health worker deficit compared to the WHO standard [6]. In response to the health workforce shortage and the increased demand for HIV treatment, in 2004 the Ugandan government developed and pilot tested community-based ART delivery and task-shifting models [6,7]. This effort included use of community distribution points and mobile units, or mixed models of community-based and facility-based service provision, to bring HIV care and treatment closer to the community with the delegation of tasks to less specialized health workers and laypersons. With a health worker shortage, these models are critical in removing barriers to accessing ART care and reducing associated costs.

Although projections of resources needed to deliver ART following the 2015 WHO guidelines have been made at the global level, such projections have not been made frequently in Uganda due to the lack of comprehensive costing data [8,9]. Several initiatives providing ART have been implemented in Uganda; however, there is little information available about the costs of providing ART across these service delivery models [10]. In addition, the available costing studies have had a broader focus on public sector health facilities or were solely based on budgeting data (projected data), without necessarily providing actual costing data [11]. To help fill this gap, we aimed to provide the descriptive annual ART cost per ART client at the three largest non-profit organizations serving PLHIV in Uganda – The AIDS Support Organization (TASO), Kitovu Mobile (KM) and Uganda Cares (UC) – using a retrospective review of routine data. The service delivery models provide free outpatient ART services and serve mainly rural and semi-urban populations [12]. The findings from this study will be valuable for budgetary efforts to ensure universal access to ART in Uganda, following the 2015 WHO guidelines. In addition, costing data is an important input for modelling cost-effectiveness and cost-efficiency analyses to promote long-term sustainability of ART in Uganda and similar contexts [13,14].

## Methods

### Description of the three service delivery models

Data were collected as part of a larger study assessing the three task-shifting and community-based ART support programmes in Uganda (Table 1) [12]. The three participating organizations serving PLHIV in Uganda included the following: TASO, which comprises 11 centres in four regions and serves nearly 100,000 PLHIV annually; KM, which operates in 10 districts in southwestern Uganda and serves about 2000 PLHIV annually; and UC, which operates 29 clinics in four regions of Uganda and serves nearly 50,000 PLHIV annually.

**Table 1:** Characteristics of the three ART delivery models (2012)

Characteristics	Kitovu Mobile	TASO Entebbe	TASO Gulu	TASO Jinja	TASO Rukungiri	UC Balikudembe	UC Nakawa Market	UC Lyantonde	UC Kalisizo
HIV prevalence (adults aged 15 to 49)	10.6%	7.1%	5.8%	5.8%	8.3%	7.1%	7.1%	8.0%	10.6%
Geography	Rural	Semi-urban	Rural	Semi-urban	Rural	Urban	Urban	Rural	Rural
Personnel									
Doctors	1	3	3	3	3	1	1	0	0.5 from MOH
Clinical officers	2	2	2	1	1	1	0	1 from MOH	0.5 from MOH
Nurses	10	5	5	5	5	6	2	1.5 from MOH	1 UC 1.5 from MOH
Lab technicians	1	3	3	2	2	0.5 shared with Nakawa	0.5 shared with St. Balikudembe	1	1
Data managers	1	3	3	3	3	0.8	1	1	1
Total	15	16	16	14	14	9.3	4.5	4.5	5.5
Number of ART clients	1387	6329	6969	5454	4062	2498	530	1250	1669
Number of ART visits	12,510	23,461	28,654	32,233	27,693	12,636	6076	10,345	11,420

Notes: Kitovu Mobile operates in one region, UC operates in four regions and TASO operates in 10 regions. HIV prevalence from Uganda DHS, Demographic and Health Survey, 2012. Personnel and ART client visits are from the organizational structure and records. ART, antiretroviral therapy; MOH, Ministry of Health; TASO, the AIDS Support Organisation; UC, Uganda Cares.

TASO delivers HIV care through its 11 service delivery centres in four regions across Uganda. Each TASO centre has two types of service delivery models: 1) TASO-Central and 2) TASO outreach clinics, called *community-based drug distribution points* (TASO-CDDPs). TASO-Central clinics provide ART services to clients recently initiated to ART, or complicated cases, as well as CD4 and viral load testing. TASO-CDDPs mainly dispense antiretroviral (ARV) drugs and provide counselling and health exams to stable clients at the community level. At TASO, doctors take on critical and complicated cases and supervise lower-level staff, including nurses and expert clients. Expert clients are PLHIV who have been trained to provide ART adherence counselling, monitor clients lost to follow-up and dispense ARVs. Nurses and expert clients mainly dispense ARVs to stable clients at the community level. In 2012, TASO served a total of 68,584 HIV patients, of whom 33.3% (22,814) were on ART.

HIV care and treatment services at KM are delivered at 111 non-facility-based community locations in 10 districts of the south-western region in Uganda. The organization employs 15 health professionals (doctors and nurses) and 177 expert clients. KM is a task-shifted model where a limited number of doctors undertake the overall management or supervisory roles and provide care to critically ill clients. Expert clients dispense ARVs and provide adherence counselling. In 2012, KM served 2007 clients, of whom 69.1% (1387) were on ART. Both CD4 and viral load testing were done at government laboratories.

UC is a collaborative partnership with the Ministry of Health (MOH) and the AIDS Health Care Foundation. The organization has been providing ART at no cost in Uganda since 2001 in 29 clinics across four regions. UC operates two types of service delivery models: 1) UC stand-alone (UC-S) clinics, which are located at UC centres; and 2) UC-MOH, which provides drugs and other supplies to MOH health facilities for HIV care. UC also practises task-shifting as a limited number of doctors take on critical cases or cases referred by nurses, whereas nurses dispense ARVs and conduct routine health assessments for stable clients. In 2012 UC served a total of 3495 HIV clients, of whom 80.3% (2807) were on ART. CD4 count was performed on site whereas viral load tests were sent to government laboratories for analysis.

### Site selection

Four TASO centres out of 11 total centres were purposively selected to ensure fair regional representation: Entebbe (Central-1), Jinja (East-Central), Gulu (Mid-North) and Rukungiri (South-West).

KM operates in only one region and all of its 111 outreach mobile units were selected. Currently, UC operates 29 clinics in four regions: North-East, Mid-East, Central-1 and Central-2. Among the four regions of operation, the study team purposively selected the following clinics to ensure a reasonably fair distribution across the regions of Uganda: 1) Soroti, 2) Nakawa, 3) Balikuddembe; 4) Maddu, 5) Rakai, 6) Lyantonde, 7) Kalisizo, 8) Mulanda and 9) Nagongera. Five UC clinics had excessive missing data and they were therefore removed from the analysis.

### Costing approach

The costing analysis was undertaken from the provider's perspective. The analysis only included costs that were directly incurred by the provider and excluded costs covered by clients. Ancillary and opportunity costs incurred by patients, such as transportation and time, were not collected.

### Data collection period

We chose to collect 2012 data in order to analyze the most recent cost data available (as the study protocol was developed in 2013). Data collection was completed between June and September 2013. The original costing data were recorded in Ugandan shillings (UGX) and converted to US dollars (USD) using 2012 historical exchange rates from OANDA.com. Monthly 2012 data from the sampled facilities were collected and then aggregated for the entire year. The total number of visits for 12 months and the total number of clients at mid-year were used in the analysis.

### Data collection methods

The ART drug costs, number of clients, number of visits and operational costs were collected retrospectively using routine monitoring and evaluation (M&E) data from the organizations. In addition, other costs related to service delivery – accounting records, client visit logs, ART distribution logs, equipment inventories and routine reports – were collected. Salaries and benefits of staff directly providing ART services were collected from payroll records. Time spent on ART service delivery was determined based on interviews with staff and their managers, as well as reviews of staff levels of effort in ART services. For staff not working full-time on ART-related services, the time spent on ART was calculated as a ratio of the total number of hours spent on ART divided by the total working hours (eight) per day. The percentage of office rent and operational costs attributed to ART was calculated based on the number of ART clients in relation to the total number of clients for the organization.

Costing data were collected from a programmatic perspective, which included all site-level costs of outpatient ART and supportive care. In addition to direct service provision costs, the study examined site administration, management and operational costs at each site.

A cost data capture tool was developed – according to the US President's Emergency Plan for AIDS Relief (PEPFAR) guidelines on cost elements to be collected for ART programmes – and administered at all three organizations [15]. The tool captured the following: 1) personnel costs, including salaries and allowances of staff who provide ART services as well as administrative staff who manage and support the clinics; 2) ARV costs; 3) other non-ART drugs and supplements, including drugs to prevent opportunistic infections, vitamins, TB drugs and nutrition support; 4) laboratory services related to ART delivery, such as CD4 count and viral load testing, TB testing and basic blood testing; and 5) operational costs, for example equipment, furniture, office rental, car rental, fuel, insurance, travel and office utilities.

### Apportionment of shared costs

Apportioning costs for staff not full-time on ART service delivery was estimated based on the number of ART patients *versus* total patients. For TASO, which has a regional structure, personnel costs at the national level were equally shared among the four TASO centres. At UC and KM, headquarter operational costs attributed to ART were equally distributed among all the service delivery points of the organization.

### Missing data

Missing data on office space rental and office expenses for UC and KM were replaced with data from the months available. This approach is considered acceptable because office and rental expenses are fairly stable from month to month. Missing data on ARVs and staff time were excessive (30% and above) at five of the nine UC sites; therefore they were excluded from this analysis to ensure accuracy. ARV cost data were complete for all sites. Staff time spent on ART services were calculated for all sites based on accounting records, project records, payroll data and interviews. TASO had complete costing data across all five cost components.

### Data analysis

Data from the data capture tool were entered into Microsoft Excel and organized into the five different cost components as described above. Direct costs of ARVs and other ART-specific commodities were captured and analyzed at face value. The total ART-related costs were divided by the total number of annual visits and total number of annual patients to estimate cost per ART visit and annual cost per ART patient.

### Ethical considerations

The study was reviewed and approved by the Population Council and the Ugandan National Council for Science and Technology IRBs. All of the costing data were extracted from accounting records that did not contain any patient-specific data or personal identifiers. The total number of patient visits in 2012 was collected from clinic records. No personal information of patients was recorded in the data capture tool.

## Results

### Estimation of ART cost per visit and annual ART cost per client

Table 2 summarizes the 2012 total annual ART-related expenditure, the cost per client visit and annual cost per client in USD. Table 2 also shows the average number of visits per client across the three models. In particular, clients at KM made an average of nine visits to the KM outreach locations for ART care, whereas TASO clients averaged five visits and UC clients averaged seven visits annually. The average cost per visit was $38 for UC, $45 for KM and $74 for TASO clients. The average annual cost per client was $404 for KM, $332 for TASO and $257 for UC clients. The average cost per client for all three organizations was $331 and varied across the four TASO centres and four UC clinics (data not shown).

**Table 2:** Average cost per visit and annual cost per client across the three models (2012)

Model	Location	Total expenditure (USD)	Total ART clients	Total ART visits	Average annual visits/client	Average cost per ART visit (USD)	Annual cost per ART client (USD)
Kitovu Mobile	Rural (Southwestern Region)	$560,756	1387	12,510	9.0	$45	$404
TASO	Rural (Gulu)	$2,094,695	6969	28,654	4.1	$73	$301
	Rural (Rukungiri)	$1,944,096	4602	27,693	6.0	$70	$422
	Semi-urban (Jinja)	$1,969,940	5454	32,233	5.9	$61	$361
	Semi-urban (Entebbe)	$1,744,231	6329	23,461	3.7	$74	$276
	Overall cost	$7,752,962	22,814	112,041	4.8	$69	$332
Uganda Cares	Urban (St. Balikudembe)	$791,009	2498	12,636	5.1	$63	$317
	Urban (Nakawa Market)	$190,222	530	6076	11.5	$31	$359
	Rural (Lyantonde)	$272,841	1250	10,345	8.3	$26	$218
	Rural (Kalisizo)	$274,717	1669	11,420	6.8	$24	$165
	Overall cost	$1,528,789	5947	40,477	6.8	$38	$257

ART, antiretroviral therapy; TASO, The AIDS Support Organisation; USD, US dollars.

### Cost distribution across the five components of each model

Table 3 summarizes cost distributions across the five cost components at all three organizations.

**Table 3:** Cost distribution across five cost components (2012)

Model		Personnel	ARV drugs	Other drugs	Laboratory	Administrative costs	Total USD
Kitovu Mobile	Total	$140,529	$260,641	$59,439	$13,150	$86,996	$560,756
	Distribution	25.1%	46.5%	10.6%	2.3%	15.5%	100%
	Per client	$101.32	$187.92	$42.85	$9.48	$62.72	$404.29
TASO	Total	$1,655,930	$3,399,418	$1,145,544	$477,737	$1,074,333	$7,752,962
	Distribution	21.4%	43.8%	14.8%	6.2%	13.9%	100%
	Per client	$72.58	$149.01	$50.21	$20.94	$47.09	$339.83
Uganda Cares	Total	$135,106	$1,007,376	$65,441	$199,366	$121,500	$1,528,789
	Distribution	8.8%	65.9%	4.3%	13.0%	7.9%	100%
	Per client	$22.72	$169.39	$11.00	$33.52	$20.43	$257.07

ARV, antiretroviral; TASO, The AIDS Support Organisation; USD, US dollars.

*ARV costs*: ARVs accounted for a significant portion of the total ART-related costs. In particular, ARVs comprised 47% of the total cost among KM clients, compared to 44% among TASO and 66% among UC clients. However, the annual ARV cost per client across these three organizations was comparable ($188 among KM, $149 among TASO and $170 among UC clients). It is important to note that in 2012, 95% of TASO clients and 98% of KM and UC clients were on first-line drugs.

*Personnel costs*: There are significant variations in personnel and other costs across the three organizations. Personnel costs accounted for 25% (KM), 21% (TASO) and 9% (UC) of the total costs. The strikingly lower personnel costs within the UC model were likely due to the fact that some government staff providing services at UC clinics were not captured. This was one limitation regarding personnel costs within the UC models and thus comparisons should be made with caution.

Other (laboratory, administrative and non-ART drugs) costs: KM spent about 15% of their total expenses on operations and overhead, 11% on non-ARV drugs and 2% on lab services. TASO's distribution of other costs was fairly similar to KM, with 15% attributed to non-ARV drugs, 14% to administrative expenses and 6% spent on lab tests. UC's distribution of other costs was much lower, with 13% of the total expenses spent on labs, 8% on operations and 4% on non-ART drugs. The differences in total ART-related costs per client per year in the three organizations are due to the differences in personnel and these other costs.

## Discussion

This study generated data on ART-related costs and resources expended to provide ART to PLHIV in Uganda. We estimated cost per outpatient ART visit and average outpatient annual ART costs using routine health service data from three non-profit AIDS service organizations representing three different ART service delivery models in Uganda. These models are considered decentralized and task-shifted. Doctors or trained clinical nurses are responsible for newly initiated ART clients and critically ill cases; other tasks including drug dispensing and routine health exams are performed by nurses and expert clients. In addition, KM provides services at the community level, TASO provides services at both the facility and community level and UC provides services mainly at the facility level. Consistent with previous costing analyses [8,10,16–20], we found that ARV-related expenses accounted for a significant portion of the total ART-related cost, followed by personnel and administrative costs. In particular, ARVs accounted for nearly 50% of the total expenses for the KM and TASO models and for nearly 70% for the UC model.

Overall, the average annual cost per ART client among the three organizations ($331) was lower than previous analyses conducted in 2008 to 2009 among five PEPFAR countries (Ethiopia, Uganda, Botswana, Nigeria and Vietnam; 2009 data), in which the median annual ART cost was $800 [10]. Another systematic review of studies conducted between 2001 and 2009 found a median ART-related cost of $792 for low-income countries [21]. However, our estimated ART costs were slightly higher than a recent study using 2010 to 2011 data that showed an average cost of $208 per client (Ethiopia, Malawi, Rwanda and Zambia) [20]. Another analysis of 8500 patients from an urban ART centre in Kampala using even more recent data (2012 to 2013) showed a comparable average annual cost per client: $218 among clients on ART for the first year, $284 for clients on ART for more than one year and $431 for patients with TB co-infection [22]. The cost of ARVs has decreased since 2010 [10,13], which is likely the largest contributing factor for the lower annual ART cost in our study and a few other recent studies [13,14]. In addition, these three participating organizations have matured and may be more efficient in serving an increased number of ART clients, as suggested by previous research [13,14]. Lower ART costs suggest that future ART programmes may become even less expensive, especially with the continuing reduction of drug costs.

Another notable finding was the variety in unit costs across sites within each model and across the three models, particularly personnel and operational costs. This finding indicates that there may be potential for cost savings for future ART programmes in Uganda. The variation reflects the differences across the service delivery models as well as the different package of services provided to patients. In particular, the cost per visit was lowest at UC and KM but significantly higher at TASO. However, cost per client was lowest for UC, followed by TASO and then KM. It is important to note that KM was found to be the most expensive model regarding annual ART cost per client. It is surprising that a more decentralized model like KM costs more than less decentralized models like UC and TASO. However, this finding is likely because KM employed a large number of expert clients and had to pay for the ART mobile units to move around rural areas where KM implements its activities. In addition, on average, KM clients made 2 and 1.5 times the total number of visits made by TASO and UC clients, respectively. It is noteworthy that the convenient access and the flexibility of ART visits (even without appointments) might explain the higher number of annual visits per client at KM. In order to achieve efficiency among community-based ART models, such as KM, the number of visits per client per year need to be reduced to four times or fewer.

The distribution of costs was quite comparable between KM and TASO, which is consistent with previous study findings [8,10,13,18,20,23]. KM employed a large number of expert clients (177) for their outreach activities, resulting in personnel costs comparable to that of TASO and higher than UC's personnel costs. However, interpretation of this finding should be made cautiously because UC employs a number of staff from the government and their salary data were not fully captured in this costing data. In addition, UC also used facilities offered by the government free of charge, resulting in a much lower operational cost compared to KM and TASO and contributing to the overall lower ART cost per client for the UC model. This ultimately affects the cost distribution within the UC model. Further, even though ART expenses for UC accounted for nearly 70% of the total cost, the total UC ART cost in terms of absolute dollar value was quite comparable with KM and TASO.

## Limitations

First, data for this study were collected retrospectively using routine accounting data, client visit logs, ART dispensing logs, staff payroll data and interviews; thus they were likely to have been subject to recall bias and other types of errors. Second, missing data is likely another threat to the accuracy of our estimate, particularly for the UC model. However, we excluded data from five of the nine UC clinics with excessive missing data. Recall bias and missing data are common limitations of routine programme data, especially data from local and grass-roots-level organizations. Third, although the percentage of clients on second-line ARV drugs was small (2% for KM, 2% for UC and 5% for TASO), we were not able to separate first-line and second-line ARV costs. Fourth, data were collected from only three non-profit HIV service organizations and therefore our cost estimates are likely not representative of ART costs among patients receiving services from other health sectors or living in other parts of Uganda. Nonetheless, these data are vital for future programming, budgeting and costing analysis and will enrich the pool of available ART costing data, which will likely improve the validity of future systematic analysis.

Although ascertaining clients’ ancillary and opportunity costs would have been important to complement the financial data, we were not able to collect information on costs such as time spent waiting at the clinic, transportation and other out-of-pocket payments. Because of data limitations, we did not focus on comparing the annual ART cost per client across the three organizations. Our main goal was to provide estimated ART-related costs per client and to enrich the pool of limited costing data available in Uganda and similar contexts. Nevertheless, these ART costing data yield valuable information for these three organizations and other AIDS organizations in Uganda and other low- and middle-income countries, to assist with future programme planning and budgeting. As we move towards treating everyone with HIV regardless of their CD4 count, careful budgeting is critical to ensure universal access to ART. In addition, despite these limitations, the study has demonstrated the feasibility of using existing routine data to estimate the cost per ART patient visit, while highlighting the need to strengthen the capacities of local organizations to better collect, document and use routine data.

## Conclusions

Our study provides the most recent available costing data from the three largest HIV service organizations, representing three different ART service delivery models in Uganda [7]. Unit costs, cost distribution and resource utilization varied widely across the three sites and models, suggesting the potential for efficiency gains in ART service delivery. In particular, HIV programmes in Uganda may save costs by reducing the number of annual ART visits to the national standard (four ART visits a year on average). Further, non-profit organizations providing ART services, similar to these three organizations, may benefit from collaborating with the government and using government facilities to reduce operational costs. Additionally, ART is evolving rapidly with lower ARV costs and the 2015 WHO guidelines recommending treatment for all PLHIV [3]. Our findings of lower annual ART costs compared to previous analyses in Uganda and the region add value to several recent estimated ART costs, suggesting that we may be able to treat more people with the same or even fewer resources. Lastly, the collection of costing data to measure unit costs, cost-effectiveness and cost-efficiency remains critical [2,4]. ART service delivery sites in country would benefit from implementing a standardized cost data-capture tool or M&E system that allows for comparison across sites. In addition, supportive supervision is critical to ensure data quality.
